# Supramolecular Structure and Mechanical Performance of κ-Carrageenan–Gelatin Gel

**DOI:** 10.3390/polym14204347

**Published:** 2022-10-15

**Authors:** Anastasiya O. Makarova, Svetlana R. Derkach, Aidar I. Kadyirov, Sufia A. Ziganshina, Mariia A. Kazantseva, Olga S. Zueva, Aidar T. Gubaidullin, Yuriy F. Zuev

**Affiliations:** 1 Kazan Institute of Biochemistry and Biophysics, FRC Kazan Scientific Center of RAS, Lobachevsky St. 2/31, 420111 Kazan, Russia; 2 Department of Chemistry, Murmansk State Technical University, Sportivnaya Str. 13, 183010 Murmansk, Russia; 3 Institute of Power Engineering and Advanced Technologies, FRC Kazan Scientific Center of RAS, Lobachevsky St. 2/31, 420111 Kazan, Russia; 4 Zavoisky Physical-Technical Institute, FRC Kazan Scientific Center of RAS, Sibirsky Tract 10/7, 420029 Kazan, Russia; 5 HSE Tikhonov Moscow Institute of Electronics and Mathematics, Tallinskaya St. 34, 123458 Moscow, Russia; 6 Department of Physics, Kazan State Power Engineering University, Krasnoselskaya St. 51, 420066 Kazan, Russia; 7 Arbuzov Institute of Organic and Physical Chemistry, FRC Kazan Scientific Center of RAS, Arbuzov St. 8, 420088 Kazan, Russia; 8 A. Butlerov Chemical Institute, Kazan Federal University, Kremlevskaya St. 18, 420008 Kazan, Russia

**Keywords:** κ-carrageenan–gelatin hydrogel, supramolecular structure, mechanical performance

## Abstract

In this work, by means of complex physicochemical methods the structural features of a composite κ-carrageenan–gelatin system were studied in comparison with initial protein gel. The correlation between the morphology of hydrogels and their mechanical properties was demonstrated through the example of changes in their rheological characteristics. The experiments carried out with PXRD, SAXS, AFM and rheology approaches gave new information on the structure and mechanical performance of κ-carrageenan–gelatin hydrogel. The combination of PXRD, SAXS and AFM results showed that the morphological structures of individual components were not observed in the composite protein–polysaccharide hydrogels. The results of the mechanical testing of initial gelatin and engineered κ-carrageenan–gelatin gel showed the substantially denser parking of polymer chains in the composite system due to a significant increase in intermolecular protein–polysaccharide contacts. Close results were indirectly followed from the SAXS estimations—the driving force for the formation of the common supramolecular structural arrangement of proteins and polysaccharides was the increase in the density of network of macromolecular chains entanglements; therefore, an increase in the energy costs was necessary to change the conformational rearrangements of the studied system. This increase in the macromolecular arrangement led to the growth of the supramolecular associate size and the growth of interchain physical bonds. This led to an increase in the composite gel plasticity, whereas the enlargement of scattering particles made the novel gel system not only more rigid, but also more fragile.

## 1. Introduction

Biomolecular hydrogels are attracting great attention as “smart” materials for different biotechnology and biomedicine applications [1,2,3,4,5,6,7].

Hydrogels form three-dimensional polymer networks capable of accumulating and holding up to 99% of water in their volume space [8,9]. Technologically more attractive are the “physical” hydrogels with the polymer network maintained by the mechanical weaving of polymer molecules and the combination of intermolecular interactions, such as ionic bridges, hydrogen bonding and hydrophobic forces [10]. Intriguingly, that supramolecular polymer network is preserved both in the gel and sol states, although with definite structural differences [11]. Because of biocompatibility, structural similarities to the native extracellular matrix and excellent permeability for nutrients and metabolites, hydrogels based on natural polysaccharides and proteins have gained considerable attention [12]. In particular, hydrogels are widely applied in tissue engineering, dressing and wound healing, where among others their mechanical properties are of great importance [13]. The development of hydrogel systems in tissue and organ regeneration, such as bones, cartilage and intervertebral discs presents a significant challenge. Engineered hydrogels have to meet certain requirements in biomedicine including definite fluidity under moderate pressure for use in injections, quick coagulation at the target site and maintenance of sufficient integrity and mechanical strength [14,15]. Thus, the mechanical performance is one of the most important properties of engineered implants.

Among natural biopolymers, biodegradable gelatin and κ-carrageenan are the universal candidates for the food and pharmacy industries and tissue engineering. Gelatin is the peptide product of partially hydrolyzed animal protein collagen [16]. Gelatin forms thermo-reversible hydrogels through the coil-to-helix conformational transition [17]. κ-Carrageenan is a linear galactan, built of repeating disaccharide units of 1,4-linked α-D-galactose and 1,3-linked -β-D-galactose with a variable proportion of sulfate groups at different positions [18]. The hydrogel formed from κ-carrageenan occurs via the entanglement of polymer chains with the formation of ordered structures in the junction domains. The gelation of κ-carrageenan is temperature-dependent, with gelation at cooling and melting upon heating.

In the scientific literature there are many different examples of the engineering of mechanical properties of hydrogels to retain their favorable biomedical characteristics. It may be the mechanical reinforcing of the hydrogel structure by fibers, for instance by collagen in molecular form or by collagen fibrils or fiber bundles [15]. Another popular approach to modulate the initial mechanical properties of the biopolymer systems is the initiation of the additional chemical and physical cross-linking [19]. Because the subject of the present study is physical hydrogels, we shall refer here only to the possibilities of physical cross-linking in the mechanical performance of hydrogels, for example, such as the artificial increase in hydrogen bonding by the physical incorporation of H-bond donors of a polymer nature [20]. One other modifying approach is the use of synthetic nanosized additivities, such as carbon nanotubes which operate as structural and mechanical modifiers of the biopolymer network [11,21]. The combination of polysaccharides and proteins is one of the additional engineering approaches that influences the gel structure, its jelling conditions and the alteration of physical–chemical properties [22]. In gelatin/carrageenan hydrogels, the synergistic effects have been determined [23], for example, the increase in mechanical strength [24], flexibility and porosity and the water retention capacity [25].

In this work, the novel information on the modulation of gelatin structure and mechanical performance in the gel state were studied. The goal of the present study was to investigate the influence of κ-carrageenan on gelatin hydrogel structure and mechanical properties below the jelling temperature. The structure was analyzed with a complex of experimental approaches including small-angle X-ray scattering (SAXS), powder X-ray diffraction (XRD) and atomic force (AFM) microscopy. The interconnection between structural changes in gelatin hydrogel and the gel mechanical properties under the admixture of κ-carrageenan were demonstrated by rheology experiments.

In the present study, we compared the structure and mechanical behavior of the initial gelatin system with the data obtained earlier for the mixed κ-carrageenan–gelatin gel [11]. For this purpose, in this work we used a part of the graphical data and numerical characteristics of the mixed system obtained and given.

## 2. Materials and Methods

### 2.1. Materials

The sample of gelatin (gel strength 300, Type A, produced by Sigma-Aldrich, St. Louis, MO, USA) from porcine skin with M_η_ = 100 kDa was used as the protein component of hydrogel. κ-Carrageenan (Sigma-Aldrich, St. Louis, MO, USA) with Mη = 430 kDa was used in experiments as the polysaccharide. All measurements were carried out in ultrapure water purified using the “Arium mini” water purification system (Sartorius, Gottingen, Germany).

### 2.2. Preparation of Solutions and Gels

Aqueous solutions of κ-carrageenan and gelatin were prepared separately. Initially, the biopolymers were swollen in distilled water at 20 °C for 15 h. Solutions of κ-carrageenan and gelatin were prepared separately and dissolved at 70 and 50 °C, respectively. Aqueous mixtures of κ-carrageenan–gelatin complexes were prepared by stirring the initial solutions using an ultrasound bath for 60 min at 50 °C to obtain an aqueous mixture with the desired ratio of κ-carrageenan–gelatin Z = 0.8 (*w/w*). The pH values of the mixture of gelatin with κ-carrageenan were in the range of 5.3–5.5. At the given pH and biopolymer ratio, there was no macrophase separation in the system [26]. To study κ-carrageenan–gelatin hydrogel in the gel state (the jelling temperature of the studied composition is about 25 °C [27]), the temperature was decreased from 40 to 14 °C. Before all experiments in the gel state the samples were stored at 14 °C for 1 h.

### 2.3. X-ray Powder Diffraction

The PXRD diffractograms of initial gelatin powder and gelatin gel were determined in the reflection mode using a MiniFlex 600 diffractometer (Kazan Federal University, Kazan, Russia) equipped with a D/teX Ultra detector (Rigaku, Akishima-shi, Japan) (CuK_α_, λ = 1.54178 Å, Ni-filter). The PXRD experiments with κ-carrageenan–gelatin gel were performed with the automatic Bruker D8 Advance diffractometer with the Vantec linear PSD (Bruker Corporation, Billerica, MA, USA) (λ CuK_α1_ 1.5406 Å). The scattering data in all experiments were collected in the reflection mode with a flat-plate samples, which were placed on the standard silicon plate (Bruker Corporation, Billerica, MA, USA) with zero diffraction and were kept spinning (15 rpm) throughout the data collection. The diffraction patterns were recorded in the 2θ range between 3° and 90° in 0.008° steps with a step time of 0.1–4.0 s. Data processing was performed with software packages EVA Version 11 (Bruker AXS: Karlsruhe, Germany) and TOPAS Version 3 (Bruker AXS: Karlsruhe, Germany) [28,29].

### 2.4. Small-Angle X-ray Scattering

The SAXS experiments were fulfilled with the Nanostar diffractometer (Bruker AXS, Billerica, MA, USA) (CuK_α_, λ = 1.5418 Å), coupled with the Gobbel mirrors optics and the HiStar 2D area detector (Bruker AXS, Billerica, MA, USA). The parameters of the diffractometer and the conditions and methods for performing experiments were similar to those used by us earlier and are described in detail in our publication [11]. The 2D scattering data processing was carried out with the SAXS program [30] and the calculations of structural parameters and simulation were performed with the SASView [31] and PRIMUS [32] programs. The studied hydrogels are described by the Gauss –Lorentz gel model [33], which well describes the scattering from physical networks, which is the characteristic of the gelatin and κ-carrageenan–gelatin gels. The SAXS response was modeled as the sum of exponential decays at low *s* values and the Lorentzian at higher values of *s* [34,35].

### 2.5. Atomic Force Microscopy (AFM)

The surface morphology of the samples was detected by atomic force microscope Titanium (NT-MDT, Zelenograd, Russia). Measurements were carried out under open air in the semi-contact mode. Silicon cantilevers NSG-10 (NT-MDT, Zelenograd, Russia) with a force constant of 3.1–37.6 Nm^–1^ and a resonant frequency of 140–390 kHz were used. The software Nova PX (NT-MDT, Zelenograd, Russia) was used to operate the microscope. The 2 μL volume of hydrogel was placed on a freshly cleaved mica surface and dried under ambient conditions. The AFM images were obtained at room temperature and processed and analyzed with the Image Analysis program (NT-MDT, Moscow, Russia).

### 2.6. Rheological Measurements

Rheological properties of the studied systems were evaluated using an MCR102 (Anton Paar, Graz, Austria) rotational rheometer with a “plate–plate” measuring system (two 50 mm diameter plates with a 0.5 mm gap between them). The temperature control of samples was fulfilled with the lower heating system and active casing both using the Peltier elements P-PTD200 (Anton Paar, Graz, Austria). The variation of given temperature was within ± 0.1 °C. Measurements were carried out in the following deformation modes: periodic oscillations at constant temperature (14 °C) with different amplitudes, ω, at a constant frequency, ω = 6.28 s^−1^, or varying frequency, γ, at constant amplitude, γ = 1%, the range was 0.9–139% and ω was 0.0671–23.8 s^−1^. To exclude the initial gelation time from the final results, all samples of gels were thermally stabilized during 60 min at 14.0 °C under a weak dynamic oscillation.

## 3. Results

### 3.1. PXRD Overview of the Hydrogel Phase State

**Figure 1:**
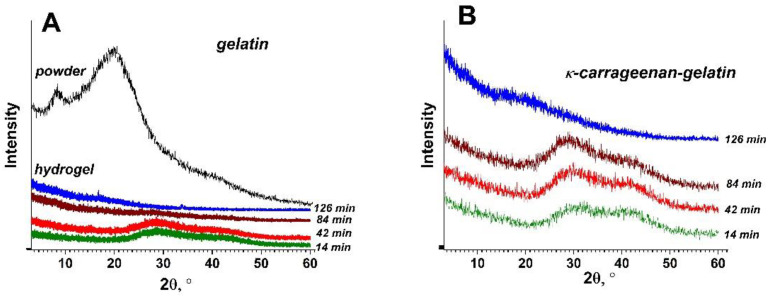
Diffraction patterns from original dry gelatin powder (black curve) and the gel state of gelatin (upon drying) (**A**), time evolution of X-ray diffraction for κ-carrageenan–gelatin hydrogel upon drying (**B**).

The powder X-ray diffraction method (PXRD) was used to compare the phase states of the initial gelatin powder product and gelatin gel under its natural drying with the structure of the κ-carrageenan–gelatin hydrogel. Original gelatin is the nanostructured sample (Figure 1A, black curve). A wide peak on the gelatin diffraction pattern at 2θ = 20° can be defined as an “amorphous halo” and corresponds to the amorphous nature of protein [25,36]. The gelatin gel state was characterized by the same type of diffraction pattern as the κ-carrageenan-gelatin hydrogel (Figure 1B), depicting two broadened amorphous halos with maximums near the 30 and 40°, which characterized the average interatomic distances. To determine the initial structure of components in the powder phase, we studied the PXRD patterns under the drying of prepared solutions. During the natural drying of the samples placed on the surface of the silicon plate, these peaks degenerated into one small, broadened peak in the interval of diffraction angles of 10–15 degrees. Its low intensity was the result of a small amount of scattering substance after the drying. It is clear that under gelatin drying, it is structured like the original powdered phase. At the same time, the κ-carrageenan–gelatin gel had the less structured state, closer to an amorphous one. It is worth noting that no crystallization of initial crystallizing components in the κ-carrageenan–gelatin hydrogel was observed, which indirectly points out the homogenization of the system without the separation of the initial components into individual associates [37].

Thus, the integration of protein with polysaccharide into a composite hydrogel suppressed their individual structure and the formation of integral supramolecular structure. The phase homogeneity of the composite hydrogel, confirmed by the PXRD method cannot exclude the aggregation and segregation processes at all size scales, which, in turn, can be estimated in a hierarchical order by SAXS and AFM experiments. The influence of such segregation on the mechanical characteristics of κ-carrageenan–gelatin gel was evaluated by studying the rheology of this system.

### 3.2. SAXS Structural Characterization of Hydrogel Sol and Gel States

**Figure 2:**
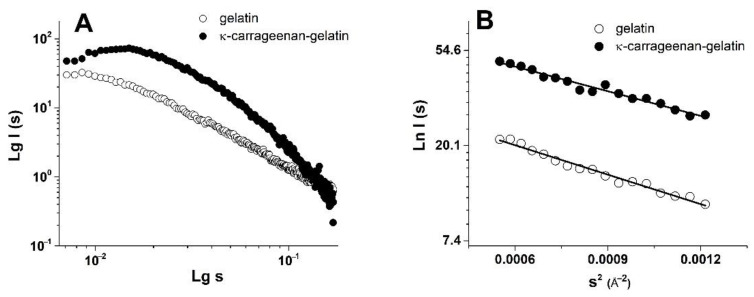
Experimental SAXS data for gelatin and κ-carrageenan–gelatin gel (**A**) and linear approximation of the Guinier plot (**B**) after background substruction.

The integration of two-dimensional experimental patterns of small-angle scattering resulted in the one-dimensional SAXS curves with the shape which is typical for systems of non-interacting particles (Figure 2). The obtained high scattering intensity is evidence of the structural microheterogeneity of the studied gels—the presence of randomly oriented scattering particles (domains of increased density), with their size corresponding to the range of SAXS techniques (1–100 nm) [38]. To analyze the morphology of the studied gels, a number of structural characteristics were calculated from the obtained SAXS experimental data.

**Figure 3:**
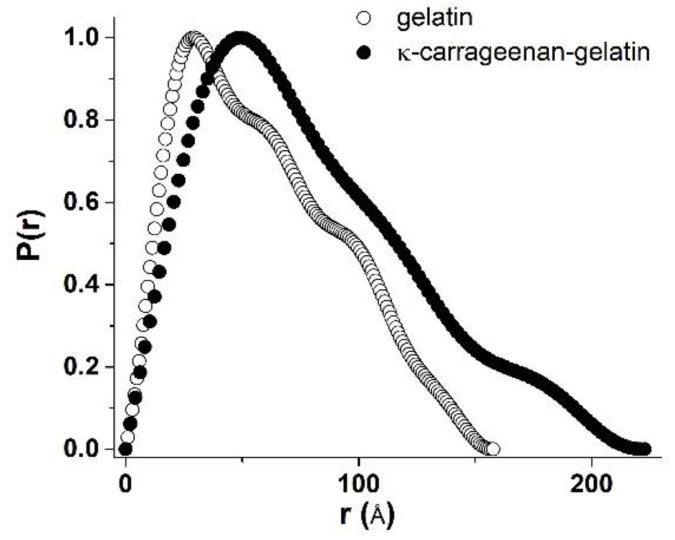
Normalized distance distribution functions *P(r)* derived from SAXS data for gelatin (open symbols) and κ-carrageenan–gelatin (filled symbols) gels.

The combined κ-carrageenan–gelatin hydrogel was characterized by more intense scattering (Figure 2A), which is associated with the participation of both the protein and polysaccharide in the common supramolecular structure. The SAXS data gave the information on the supramolecular organization of the gel-forming protein and the protein–polysaccharide systems, namely, on the size of static inhomogeneities in the studied gel structures. To analyze the morphology of studied systems, a number of parameters and dimensional characteristics were calculated on the basis of the experimental SAXS data. One of the important characteristics obtained from the SAXS experiment was the particle gyration radius *R_g_* [38]. This parameter is the rms distance of all scattering sources from the center of particle. The *R_g_* value can be used to estimate the size of macromolecular or supramolecular aggregates. The particle gyration radius *R_g_* was determined in two ways. The first one was based on the use of Guinier approximation, which is valid under the condition (*sR_g_*) < 1.3 (Figure 2B, Table 1). The second way was to estimate *R_g_* from the distance distribution function *P(r)* (Figure 3). Thus, assuming the spherical shape of particles, it was possible to calculate the effective average particle radius *R_sph_* from *R_g_* (Table 1) using the equation *R_sph_* = √(5/3) *R_g_* [38].

**Figure 4:**
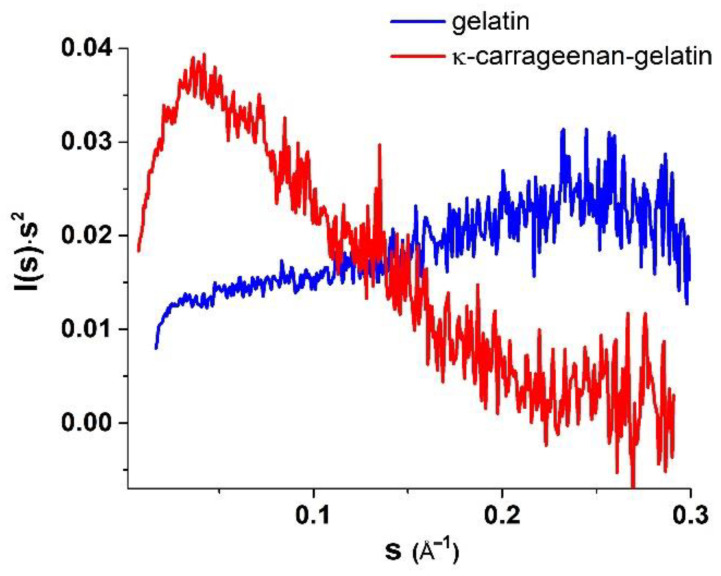
Kratky plots for gelatin and κ-carrageenan–gelatin gels.

Quite indicative for the determination of the studied supramolecular structure and the morphology is the character of the Kratky plots (Figure 4), which are also widely used to characterize the structure of hydrogels [39]. The shape of the depicted curves is a qualitative estimation of the folding or compactness of macromolecules. The bell-shaped Kratky plots (Figure 4) characterize the scattering pattern from a system of densely folded globules. Obviously, the obtained Kratky plots are also evidence of the joint participation of individual components in formation of common supramolecular structure of combined gel. With all this going on, the curve shape of the *P(r)* functions provides information about the particle shape and gives an alternative way to determine *R_g_*. In this case, the radius of gyration *R_g_*, determined using the *P(r)* curve turned out to be a much more stable estimate for the presence of admixture of polydisperse particles than the *R_g_* value from the data presented as the Guinier plot.

**Figure 5:**
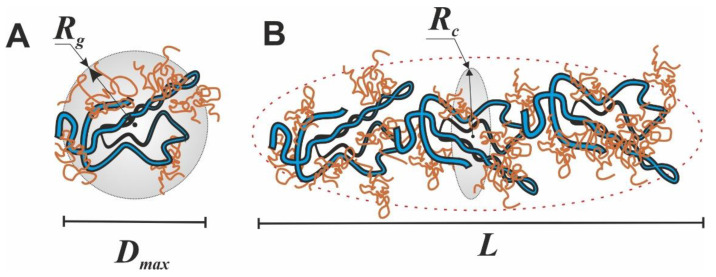
Structural schemes of spherical (**A**) and cylindrical (**B**) models indication of determined parameters.

In our previous fundamental work on the SAXS study of κ-carrageenan–gelatin hydrogels, which was referred to above, we substantiated two options for estimating the shape and size of scattering particles. Based on the obtained X-ray scattering data, we supposed two options for calculating the morphology of these hydrogels. The first one is the use of the spherical symmetry model of formed aggregates, when the radius of such spherical particles is quite unambiguously calculated from the obtained *R_g_* values of particles. However, the comparison of obtained *R_g_* values with the calculated maximum distances *D_max_* in particles (Table 1) characterizes the shape of particles as noticeably deviating from spherical, i.e., an elongated (cylindrical) shape (Figure 5).

Additional confirmation of this assumption is presented in our other article [17]. On the base of FTIR spectroscopy and molecular docking it was proposed that in the κ-carrageenan–gelatin hydrogels the most energetically favorable configuration of junctions was the interaction of the gelatin triple helix segments and the κ-carrageenan double helix. Both components of the composite hydrogel and their junctions formed the rod-shaped and rigid local structures, resulting in an anisotropic structural network, which can be hypothetically imagined as elongated particles between nodes. Such a highly anisotropic system can be depicted as the ensemble of cylindrical particles with an average length *L* and cross section *r_c_*, which can be obtained from the experimental value of the gyration dimension of the cross-section *R_c_* using the modified Guinier plots (ln(*s*∙I(*s*)) vs. *s*^2^) [38]. In the special case of anisometric particles, the two-dimensional analogue of gyration radius *R_g_* is called the cross-section gyration radius *R_c_* [38]. These parameters are also presented in Table 1. Note that for a gel sample based on pure gelatin, it was not possible to identify sections on low-angle curves that would be suitable for calculating the cross-section gyration radius *R_c_*. Moreover, this is true, since pure gelatin gel is characterized by the formation of particles that are close to spherical, and only the addition of κ-carrageenan leads to the formation of elongated cylindrical particles in the mixed composition.

The analysis of the SAXS analysis shows that the admixture of κ-carrageenan to gelatin resulted in the formation of an integral supramolecular structure, where the determinative role was played by more sizable and flexible κ-carrageenan molecules. The initial gelatin gel has an amorphous nature, which can only be analyzed within spherical models. The formed integral structure of a combined polysaccharide–protein gel was characterized by elongated (cylindrical) scattering particles (domains with increased density), giving an increase in characteristic sizes independently of the used model.

### 3.3. AFM Study of Gelatin and κ-Carrageenan–Gelatin Gels

**Figure 6:**
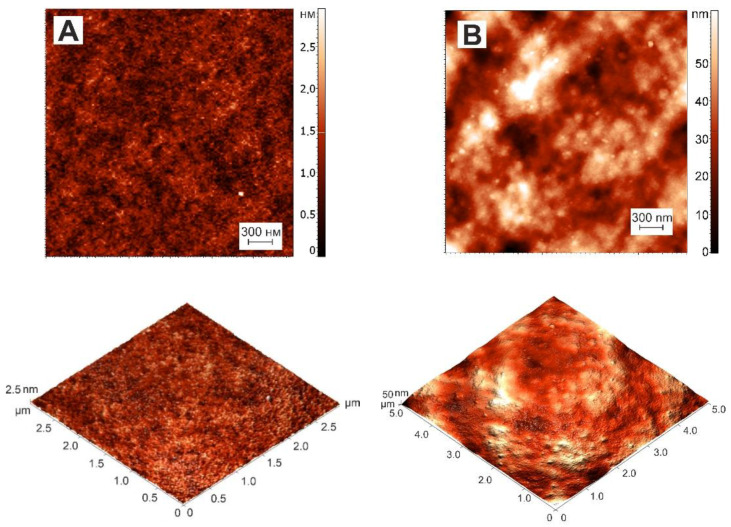
AFM pictures of xerogel (dried hydrogel) films and corresponding 3D surface of gelatin (**A**) and κ-carrageenan–gelatin (**B**) systems.

The AFM experiments [40] gave structural visualization of polysaccharide–protein hydrogels at the nanoscale. Figure 6 (A, B) depicts the AFM images of the surface of gelatin and κ-carrageenan–gelatin xerogel films. The xerogel was obtained by the drying of hydrogels, which was necessary for AFM experiment. Upon the drying of the hydrogels one can see (Figure 6) the formation of a biopolymer network or the biopolymer scaffold [41]. As can be seen from the presented data, gelatin has a smooth surface with a roughness value of *R_q_* = 0.36 nm. The admixture of κ-carrageenan to gelatin led to a change in the supramolecular structure, which is represented by an inhomogeneous rough surface consisting of densely packed spheroidal particles, and the root-mean-square value of the surface roughness of κ-carrageenan–gelatin *R_q_* = 10.7 nm. Thus, the AFM results also confirmed the formation of a common supramolecular structure in the protein–polysaccharide system.

### 3.4. Rheological Characterization of Gelatin and κ-Carrageenan–Gelatin Gels

**Figure 7:**
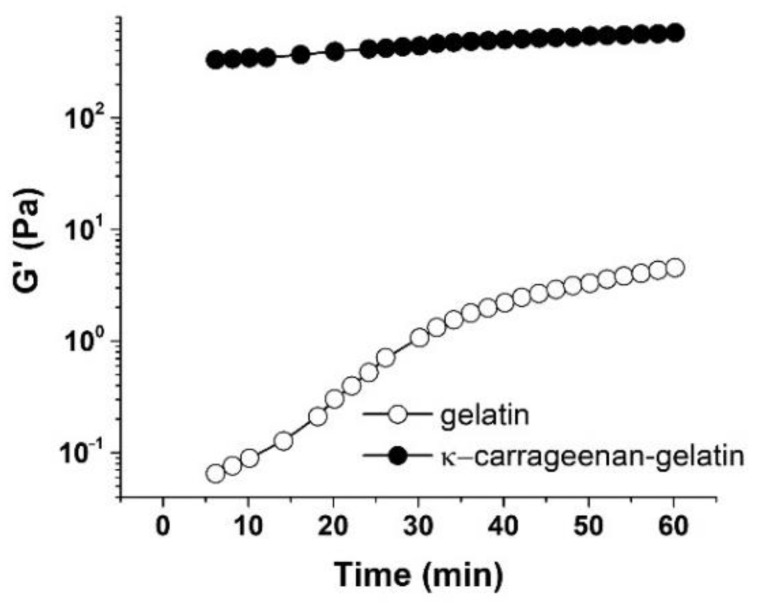
Time dependence of elastic modulus (G’) of gelatin (–) and κ-carrageenan–gelatin (,) hydrogels at 14 °C and ω = 0.2 s^−1^, deformation amplitude γ = 0.2%.

The rheological study of gelatin and κ-carrageenan–gelatin gels was used to look at the kinetics of structure formation in these systems (Figure 7). The experiments consisted of measuring the isothermal evolution of dynamic modulus of elasticity G’ at a constant frequency ω = 0.2 s^−1^ and γ = 0.2% until the stability of the data (elastic modulus) was achieved, which means the sample gelation.

The experiments were performed at a temperature of 14 °C, i.e., under the melting point of gels [27]. It will be shown below that the elasticity modulus at this temperature was practically independent of frequency, so that we were dealing with a highly elastic state of studied materials. Furthermore. the measured amplitude dependence of elasticity modulus was absent (see below), so that the corresponding modulus values refer to the region of linear viscoelasticity, and the measurement results refer to the structure of systems not destroyed by mechanical action. Therefore, the measured modulus values can be considered as analogous to the equilibrium values of the Ge modulus. Then, according to Flory’s theory [42], a semi-quantitative estimation of average degree of polymerization between neighboring grid nodes *Z_c_* is expressed as

$$Z_{c}=\frac{cRT}{G_{e}M_{n}}$$

where *c* is the polymer concentration with a weight-average molecular mass *M_n_*, *R* is the universal gas constant and *T* is absolute temperature. Then, as follows from this formula, with an increase in the elastic modulus the length of chain segment between the mesh nodes decreases, i.e., the number of nodes increases. In the case of a mesh, knots should be understood not as chemical bonds (as in rubbers), but as secondary bonds of various types and, possibly just intermolecular entanglements (entanglements). We obtained the estimated values of *Z_c_* 5.96 and 0.4 for gelatin and κ-carrageenan–gelatin gels, correspondingly. The introduction of polysaccharide to protein led to a decrease in *Z*, i.e., to increase in the grid nodes number. As a consequence, these knots prevented the usual chain folding effect observed for high-molecular compounds—(in our case, the helicalization of the gelatin chain also decreased, which was confirmed by IR spectroscopy [17]. This led to the apparent chain length becoming longer in the composite κ-carrageenan–gelatin gel. This directly correlated with an increase in the chain parameters determined by the SAXS method.

Figure 7 shows the measured time-dependence of the elastic modulus G’ for the gelatin and κ-carrageenan–gelatin systems. The kinetics of the gelation process were estimated from the time-dependence of the elastic modulus G’ (Figure 7) [43]. The gelation of gelatin occurred gradually, since there was a distinct time-dependence of the elastic modulus G’ on time (Figure 7), similar to how it is described in [44]. In contrast, the elasticity model in the κ-carrageenan–gelatin system almost instantly reached equilibrium values, which then changed very little with time, i.e., in this case, a fairly rapid formation of the structural gel network took place.

**Figure 8:**
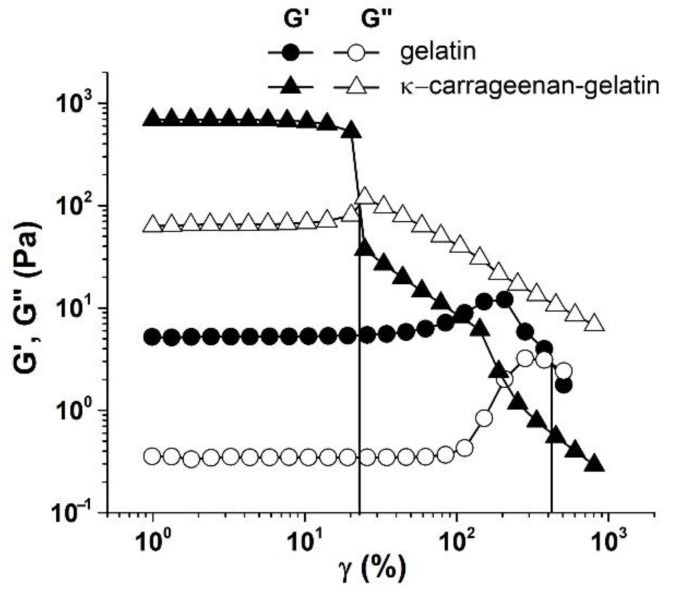
Amplitude dependences of elastic (G’) and loss (G’’) moduli for gelatin (gel) (,, –) and κ-carrageenan–gelatin (7, 8) at T = 14°C and ω = 6.28 s^−1^.

The difference between the two compared systems was clearly manifested when the elastic G’(ω) and loss G’’(ω) moduli were measured in a wide range of strain amplitudes (Figure 8). It follows from the data obtained that at small amplitudes the elastic modulus G’ confidently revealed a linear region of the mechanical behavior of gels [45], i.e., there was a fairly wide area of deformations in which the studied gels behaved like solid media. At the same time, this behavior is typical both for the κ-carrageenan –gelatin system and pure gelatin without polysaccharide additivity.

Consideration of the amplitude-dependence of modulus components made it possible to determine the limits of linear viscoelasticity, i.e., the range of strain values in which the structure of test sample was not destroyed under the action of deformation [46]. Data presented in Figure 8 allowed the estimation of strain amplitude, γ*, (and stress, σ*, as the product of the elastic modulus value and corresponding strain) corresponding to the linearity boundary. At a threshold value of amplitude exceeding a certain critical value γ*, there was a sharp drop in the elastic modulus G’ and an increase in the loss modulus G’’. This is a classic picture of destruction of gel structure, leading to transition of hydrogel from a solid to a fluid state [47,48], i.e., this point corresponded to the yield strength of the material, which can be considered as a viscous–plastic medium [49].

As one can see from Figure 8, the gelatin gel retained the linearity of the mechanical properties up to the deformation amplitude γ* ≈ 370%, while for the κ-carrageenan –gelatin gel, the transition to a fluid state had occurred already at γ ≈ 20%. Thus, the introduction of κ-carrageenan to gelatin not only promotes an increase in elastic G’ and loss G’’ moduli, but also leads to a sharp decrease in the limit of linear viscoelasticity region γ*. This means that the interaction of gelatin with κ-carrageenan provides the appearance of not only a more rigid but also a more fragile structural network in the gel. These results correlate with our observations that indicate that a change in the rheological properties of κ-carrageenan –gelatin gels occurs when the polysaccharide/protein mass ratio exceeds *Z* = 0.1, which is due to changes in the interactions in the structural network of gel.

**Figure 9:**
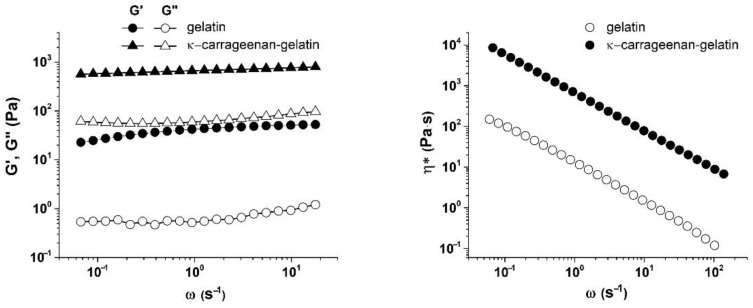
Frequency dependences of A: elastic (G’) and loss (G’’) moduli and B: complex viscosity (η*) of gelatin and κ-carrageenan-gelatin gels at T = 14 °C and γ = 1%.

Figure 9 depicts the frequency-dependence of the elastic G’ and loss G’’ moduli and complex viscosity (η*) for the systems under study. A weak dependence of G’ and G’’ on frequency was found for both systems, which, as was already mentioned above, corresponds to the pronounced solid-like behavior of gels as viscous–plastic media at low stresses. This is also associated with the higher values of the elastic modulus compared to the loss one (G’ > G”) in the entire frequency range. This behavior of studied gels is typical for soft materials with a gel-like structure, which are the viscous–plastic media with a relatively low limit [50,51]. The elastic modulus G’ of κ-carrageenan–gelatin exceeded the elastic modulus G’ of gelatin, which indicates an increase in the gelling properties of gelatin when it is complexed by a polysaccharide [52,53,54].

It can be seen from the obtained results that as the frequency increased, the complex viscosity decreased. Such dependences correspond to a situation when the systems under study exhibit non-Newtonian behavior with an increase in the shear flow velocity. The complex viscosity of hydrogel based on gelatin was an order of magnitude lower than that of hydrogel based on κ-carrageenan and gelatin. According to the considerations expressed in [55], the increase in complex viscosity indicates an increase in the density of the network of macromolecular chains entanglements; therefore, an increase in the energy costs was necessary to change conformational rearrangements within the system, which corresponds to the non-Newtonian behavior of the medium [56]. This conclusion regarding the increase in the density of network links (decrease in average distance between nodes) corresponds to the results presented above in Figure 7.

## 4. Discussion

**Figure 10:**
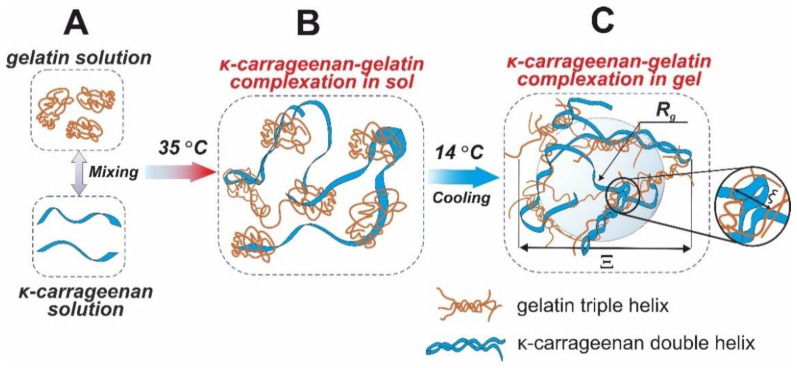
Structural schemes of κ-carrageenan and gelatin initial conformation (**A**), structural details of κ-carrageenan and gelatin complexation (**B**) and supramolecular polysaccharide–protein hydrogel (**C**).

Previously, we proposed a mechanism of interaction and the structure of the κ-carrageenan–gelatin polyelectrolyte complex [57]. The methods used in this work were aimed at obtaining information about the magnitude of static inhomogeneities in the systems under study. As the main source of quantitative and numerical information on a particular subject of the study, we used the philosophy and techniques of the SAXS method, in accordance with the scheme and dimensional definitions presented in Figure 10.

The inhomogeneities of colloidal size (1–100 nm) in gelatin and κ-carrageenan gels were studied using the SAXS method. As such, density inhomogeneities with their specific dimensions we considered as the structural units of the κ-carrageenan–gelatin system shown in Figure 9 and Figure 10. To our knowledge, the main structural element of this system is a polysaccharide–protein polyelectrolyte complex (PEC). Previously, one of our co-authors [45] showed that at the used weight ratio of κ-carrageenan–gelatin (0.8) there are no free protein or polysaccharide molecules, but almost all of them are bound in paired polyelectrolyte complexes. Their size and averaged spatial arrangement can be characterized by the parameters shown in Figure 10B. They are the PEC radius of gyration (*R_g_*), the PEC maximum dimension (*D_max_*), Ξ is the characteristic mean size of the static heterogeneities in the system and ξ is the correlation length of intramolecular interactions between the fluctuating chains of polymer, i.e., some zones of intramolecular hardening [33]. The main distinction of the system in the gel state consists in the presence of connectors (or crosslinking) (Figure 10C), combining individual PECs into a spatial 3D network via the complexation of gelatin triple helixes with one chain or a double helix of κ-carrageenan molecules.

The digital estimation of the polysaccharide–protein PEC spatial structure is presented in Table 1. One can see that admixture of polysaccharide to protein resulted in an increase in the apparent size of the scattering particles. Another deduction from the obtained SAXS results is the strong probability of the elongation of scattering of the structural elements of the newly formed hybrid κ-carrageenan –gelatin hydrogel. The results of the mechanical testing of the initial gelatin and engineered κ-carrageenan –gelatin gel showed the substantially denser parking of polymer chains in the composite system due to the huge number of new close intermolecular contacts between the polysaccharide and protein, such as the hydrophobic interaction, electrostatic ion pairing and hydrogen bonding [40]. Obviously, the close result indirectly followed from the SAXS estimations—the driving force for the formation the common supramolecular structural arrangement of protein and polysaccharide was the increase in the density of network of macromolecular chains entanglements; therefore, an increase in the energy costs necessary to change the conformational rearrangements within the system. This increase in the macromolecular arrangement led to increase in supramolecular associate size and the growth of interchain physical bonds. From the one side, the increase in network density led to an increase in the composite gel plasticity, and from another one the enlargement of scattering particles, making the novel gel system not only more rigid, but also more fragile.

## 5. Conclusions

Gelatin is one of the important hydrocolloids that is actively used in modern food and pharmacy technologies. One of the widely distributed methods for altering the mechanical properties of gelatin is the admixture of polysaccharide to gelatin systems. In this study we studied the supramolecular structure and mechanical performance of κ-carrageenan–gelatin gel in comparison with the initial gelatin one.

The presented experiments offer an overview of the structural and mechanical properties of the nanocomposite κ-carrageenan –gelatin hydrogel. The obtained results indicated that the combination of SAXS, powder X-ray diffractometry, AFM microscopy and rheology completely characterized the structure and properties of a protein–polysaccharide hydrogel. The phase homogeneity of the studied systems was confirmed by the PXRD approach. Using the SAXS method, it was shown that morphological structures based on individual components do not form in protein –polysaccharide hydrogels. The rheological study of gelatin and κ-carrageenan–gelatin gels was used to look at the kinetics of structure formation in these systems. The results of the mechanical testing of the initial gelatin and the engineered κ-carrageenan –gelatin gel confirmed the substantially denser parking of polymer chains in the composite system, obviously due to a significant increase in the intermolecular protein–polysaccharide contacts. A close result indirectly followed from the SAXS estimations—the driving force for the formation of the common supramolecular structural arrangement of the protein and polysaccharide was the increase in the density of the network of macromolecular chains entanglements; therefore, an increase in the energy costs was necessary to change the conformational rearrangements of the system. This increase in the macromolecular arrangement led to an increase in the supramolecular associate size and the growth of interchain physical bonds. This led to an increase in the composite gel plasticity, whereas the enlargement of scattering particles made the novel gel system not only more rigid, but also more fragile.

The obtained information on the structural and mechanical performance of hydrogels will open up the possibility of developing new materials with improved properties for targeted applications in biomedicine, biotechnology, as well as in any other relevant areas.

## Figures and Tables

**Table 1:** Structural parameters for hydrogels at 14 °C.

Sample	*Guinier Analysis*					*P(r) Analysis*	
	*R_g_*, Å	*R_sph_*, Å	*R_c_*, Å	*r_c_*, Å	*L*, Å	*R_g_*, Å	*D_max_*, Å
gelatin	47.5	61.3	–	–	–	42.5	149
κ-carrageenan-gelatin	56	72.3	21.2	27.4	179.5	63.4	222.7

## Data Availability

The data in this study are available on reasonable request from the corresponding author.
